# Ambient black carbon particles reach the fetal side of human placenta

**DOI:** 10.1038/s41467-019-11654-3

**Published:** 2019-09-17

**Authors:** Hannelore Bové, Eva Bongaerts, Eli Slenders, Esmée M. Bijnens, Nelly D. Saenen, Wilfried Gyselaers, Peter Van Eyken, Michelle Plusquin, Maarten B. J. Roeffaers, Marcel Ameloot, Tim S. Nawrot

**Affiliations:** 1 Centre for Environmental Sciences, Hasselt University, Agoralaan Building D, 3590 Diepenbeek, Belgium; 2 Biomedical Research Institute, Hasselt University, Agoralaan Building C, 3590 Diepenbeek, Belgium; 3 Centre for Surface Chemistry and Catalysis, KU Leuven, Celestijnenlaan 200F-box 2461, 3001 Leuven, Belgium; 4 Department of Obstetrics, East-Limburg Hospital, Schiepse Bos 6, 3600 Genk, Belgium; 5 Department of Public Health and Primary Care, KU Leuven, Herestraat 49-box 706, 3000 Leuven, Belgium

**Keywords:** Public health, Reproductive biology, Ecotoxicology

## Abstract

Particle transfer across the placenta has been suggested but to date, no direct evidence in real-life, human context exists. Here we report the presence of black carbon (BC) particles as part of combustion-derived particulate matter in human placentae using white-light generation under femtosecond pulsed illumination. BC is identified in all screened placentae, with an average (SD) particle count of 0.95 × 10^4^ (0.66 × 10^4^) and 2.09 × 10^4^ (0.9 × 10^4^) particles per mm^3^ for low and high exposed mothers, respectively. Furthermore, the placental BC load is positively associated with mothers’ residential BC exposure during pregnancy (0.63–2.42 µg per m^3^). Our finding that BC particles accumulate on the fetal side of the placenta suggests that ambient particulates could be transported towards the fetus and represents a potential mechanism explaining the detrimental health effects of pollution from early life onwards.

## Abstract (web summary)

Exposure to air pollution during pregnancy has been associated with impaired birth outcomes. Here, Bové et al. report evidence of black carbon particle deposition on the fetal side of human placentae, including at early stages of pregnancy, suggesting air pollution could affect birth outcome through direct effects on the fetus.

## Introduction

Fetal development is a critical window of exposure-related susceptibility because the etiology of diseases in adulthood may have a fetal origin and may be attributed to adverse effects of in utero environmental exposures. This causality concept is known as the Developmental Origins of Health and Disease or Barker hypothesis^1^. Ambient outdoor air pollution exposure is such a detrimental environmental factor that has been identified in this context^2,3^. Various studies have already described associations between prenatal ambient air pollution exposure and impaired birth outcomes^4^. For instance, combustion-related PM, including BC, is associated with lower birth weight^5,6^, preterm birth^7,8^, and intrauterine growth restriction^9,10^. Up to now, it remains unclear how exactly adverse effects are provoked in the fetus but various potential mechanisms have been proposed including both indirect (e.g., intrauterine inflammation) and/or direct (e.g., particle translocation) manners^11–13^.

Numerous studies have indisputably demonstrated that particulate inhalation results in health problems far beyond the lungs^14^. For example, research in different areas of the world with high^15^, moderate^16^, and low^17^ ambient PM showed that long-term exposure to particulate air pollution impedes cognitive performance. Accordingly, Maher et al.^18^ could detect the presence of combustion-derived nanoparticles from air pollution in the frontal cortex of autopsy brain samples. More importantly, the latter was one of the first studies that provided evidence of particle translocation in humans. Recently, we found BC particles from ambient air pollution in the urine of healthy children^19^, showing the ubiquity of this environmental contaminant having the potential to reach various organ systems. Appropriately, the question arises in which distant organs, such as the placenta, the particles originating from the systemic circulation might deposit. The placenta is a temporary organ that presents a natural barrier between mother and fetus during the entire pregnancy. While it was first considered to be an impenetrable barrier for xenobiotics, it has been shown that several environmental pollutants like alcohol and therapeutics can cross the placenta^20,21^. In recent years, studies were conducted to investigate whether (nano)particles can pass the placental barrier. However, these investigations are limited to in vitro cell cultures, ex vivo models and animal studies^22,23^. Hence, particle translocation to the human placenta following inhalation under real-life conditions is insufficiently studied while being essential in understanding the effects on fetal health^24^.

Here, we postulate that BC particles are able to translocate from the mothers’ lungs to the placenta. Within this framework, we employ our recently established method for detecting carbonaceous particles based on the non-incandescence related white-light (WL) generation under femtosecond pulsed illumination^25^. The study is performed on a subset of term placentae from mothers enrolled within the ENVIR*ON*AGE birth cohort study and on preterm placentae from spontaneous terminated pregnancies. We report the presence of BC particles at both the fetal and maternal side of all screened term and preterm placentae. The carbonaceous nature of those particulates and their placental embedment to preclude external contamination are confirmed. In addition, the BC load from the term placentae is positively associated with the residential BC exposure of the mothers during gestation. These results suggest that ambient particulates can be transported through the placental barrier towards the fetus, even during early and vulnerable stages of pregnancy. Hence, it strengthens the hypothesis that direct effects induced by the presence of ambient combustion-related particulates are at least partially responsible for observed detrimental health effects from early life onwards.

## Results

### Experimental protocol for BC detection in placentae

**Fig. 1:**
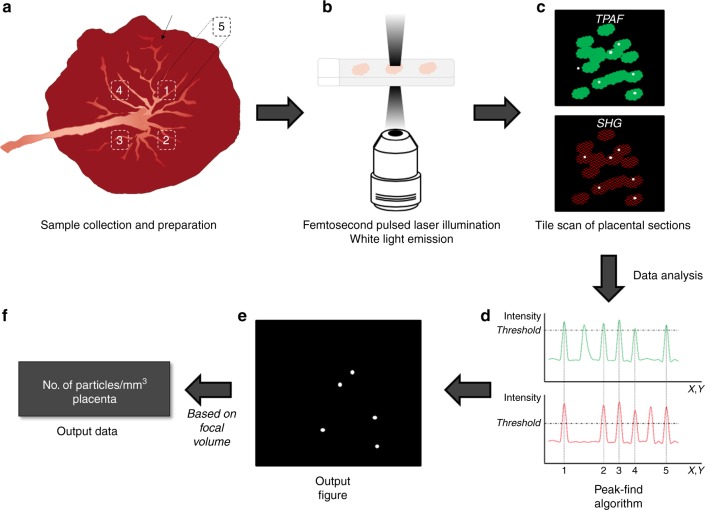
Flowchart of the experimental protocol for BC detection in the placenta. **a** Five biopsies are taken in total, of which four on the fetal side of the placenta at distinct positions oriented according to the main blood vessel (black arrow), and one (biopsy 5) at the maternal side of biopsy 1. After sample collection, the biopsies are embedded in paraffin and sections of 4 µm are prepared. **b** The placental sections are illuminated using a two-photon femtosecond pulsed laser tuned to a central wavelength of 810 nm (10 mW radiant power at the sample). **c** The WL produced by the BC naturally present in the tissue (white dots) is detected along with the simultaneous generation and detection of two-photon excited autofluorescence (TPAF) of the cells (green) and second harmonic generation (SHG) from collagen (red) (see materials and methods for detailed protocol). A tile scan of 10 × 10 images is acquired resulting in a field of view of 9000 × 9000 µm^2^ with a 12960 × 12960 pixel resolution (0.694 × 0.694 µm^2^ pixel size) and a pixel dwell time of 2.51 µs. Three different locations per placental section are imaged. **d** The number of BC particles in the obtained images is determined using a peak-find algorithm, which counts connected pixels above a certain threshold value, i.e., 0.5% and 45% lower than the highest intensity value of the TPAF and SHG pictures, respectively. **e** BC particles (white dots) in the output figure are defined as the saturated pixels found in both channels, i.e., TPAF and SHG. **f** Finally, based on the image volume estimated from the point spread function of the optical system, the result is expressed as the total relative number, i.e., the number of detected BC particles per cubic millimeter placenta

We used our previously established technique based on the WL generation of carbonaceous particles under femtosecond pulsed laser illumination^25^ to study the presence and location of BC particles in human placenta (Fig. 1). Under femtosecond pulsed illumination, placental tissue generates various label-free signals including second harmonic generation (SHG) and two-photon excited autofluorescence (TPAF). The SHG signal is originating from collagen type I while the TPAF emanates from various structures, e.g., placental cells, elastin and red blood cells. Both the SHG and TPAF signals were generated simultaneously but spectrally separated and detected. The present BC particles were analyzed based on two of the characteristic WL features: (i) the emission signals saturate compared to the TPAF and SHG, which allows thresholding of the particles in each of the two detection channels, i.e., TPAF and SHG, and (ii) the emitted WL ranges over the whole visible spectrum, so the thresholded particles should be present in both channels. A flowchart of the employed protocol is depicted in Fig. 1. Every step was designed and monitored to preclude any possible contamination.

**Fig. 2:**
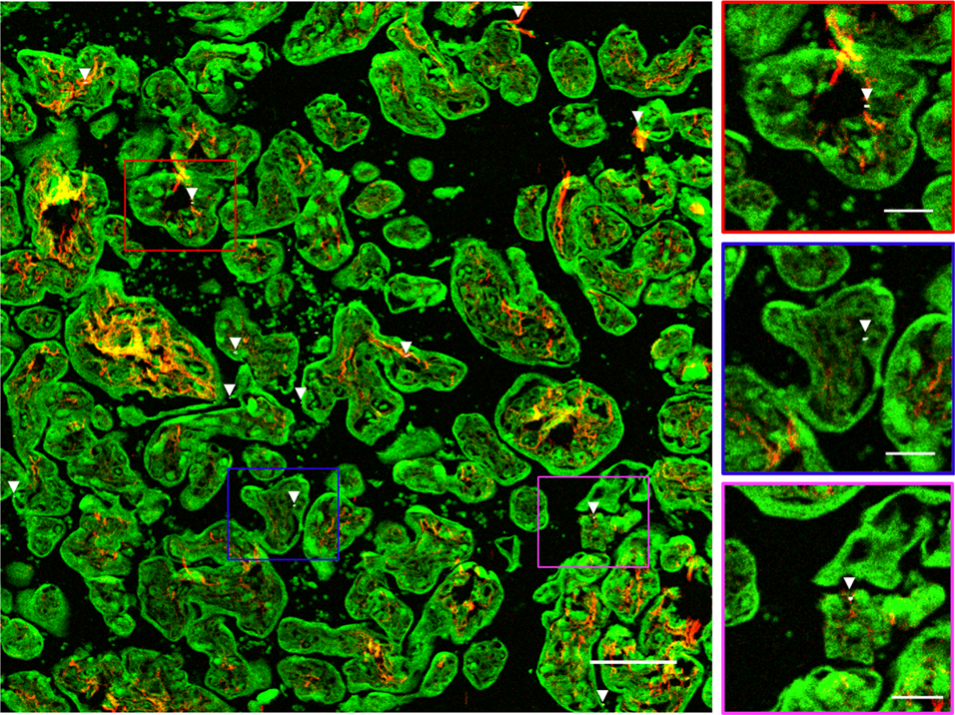
Evidence of BC particles at the fetal side of the human placenta. WL generation originating from the BC particles (white and further indicated using white arrowheads) under femtosecond pulsed laser illumination (excitation 810 nm, 80 MHz, 10 mW laser power on the sample) is observed. Second harmonic generation from collagen (red, emission 400–410 nm) and TPAF from placental and red blood cells (green, emission 450–650 nm) are simultaneously detected. Scale bar: 100 µm. The boxes on the right show the black carbon particles present in placental tissue at higher magnification. Scale bar: 30 µm

We were able to detect BC particles from ambient air pollution present in human placentae in a label-free and biocompatible manner (Fig. 2).

### Validation experiments of WL from BC in placentae

**Fig. 3:**
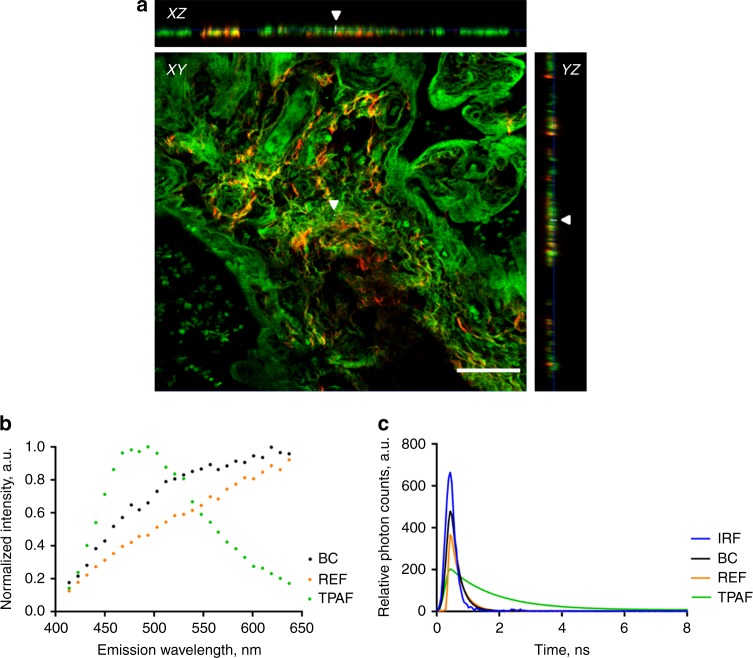
Validation experiments to confirm the carbonaceous nature of the identified particles inside the placenta. **a** XY-images acquired throughout a placental section in the *z*-direction and corresponding orthogonal XZ-projections and YZ-projections showing a BC particle (white and indicated by white arrowheads) inside the tissue (red and green). Scale bar: 50 µm. **b** Emission fingerprint of identified black carbon (BC), reference carbon black (REF) particles and two-photon excited autofluorescence (TPAF) under femtosecond pulsed illumination. **c** Temporal response of BC and REF particles and TPAF measured by time-correlated single photon counting. The instrument response function (IRF) overlaid in blue. Source data are provided as a Source Data file

Various validation experiments were performed (Fig. 3). While we strived to avoid external contamination of the placenta tissues by applying strict experimental guidelines (see details in the methods section), we checked the embedment of the BC particles inside the placental tissue. Optical sectioning in the Z-direction throughout the placental tissue and the corresponding orthogonal projections (Fig. 3a, Supplementary Fig. 3) showed that the detected BC particles are embedded in the tissue and are therefore not originating from external pollution. Subsequently, the carbonaceous nature of the identified BC particles was studied. Experiments were conducted to confirm the characteristic features of the emitted WL, which were checked for specificity and sensitivity in our previous studies^19,25^. First, we know that carbonaceous particles, including the environmental pollutant BC and commercially engineered carbon black (CB), under femtosecond pulsed near-infrared illumination generate WL that stretches over the whole visible spectrum and is extremely bright and thus saturates easily the detection channel. The emission fingerprint of the identified BC particles was recorded (Fig. 3b), which shows that indeed the signal ranges over the various emission wavelengths. The WL signal of commercially CB particles was measured as a reference, confirming the WL emission profile. In contrast, the emission fingerprint of the background signals of the placenta tissue consists of a distinct peak that does not continuously range over all wavelengths (Fig. 3b). Second, the temporal responses of the determined BC particles, reference CB particles and background TPAF (Fig. 3c) were recorded to be 250, 320, and 1400 ps, respectively. The temporal response of the BC particles is non-resolved from the instrument response function. These results are consistent and validate the carbonaceous nature of the BC particles as their temporal response is known to be instantaneous.

### Intravariability and intervariability of BC load in placental tissue

Subsequently, to evaluate the intravariability (within one biopsy) and intervariability (between different biopsies) of the placental tissue, the routinely collected biopsies from three mothers enrolled in the ENVIRonmental influence *ON* early AGEing (ENVIR*ON*AGE) birth cohort study were screened for their BC load (Supplementary Fig. 4). No significant difference in BC load was observed between the four fetal sided biopsies for the three screened mothers. On the other hand, significant differences between the fetal and maternal sided biopsies could be seen. The summary of these findings can be seen in Supplementary Fig. 4.

### Placental BC load and residential exposure during pregnancy

**Fig. 4:**
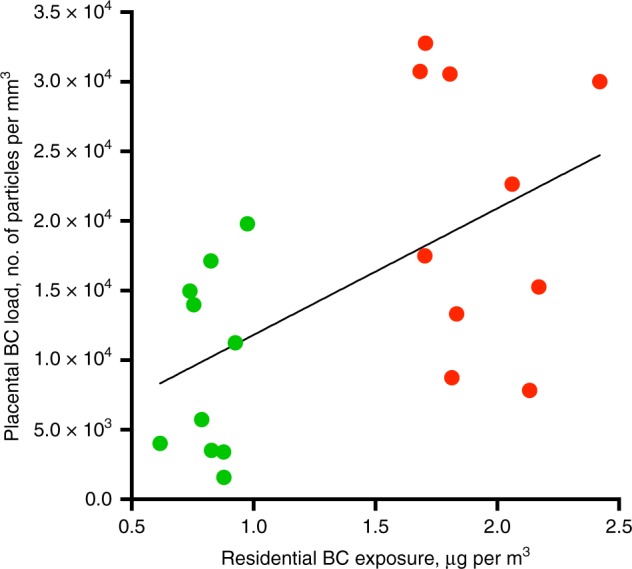
Correlation between placental BC load and residential BC exposure averaged over the whole pregnancy. The line is the regression line. Green and red dots indicate low (*n* = 10 mothers) and high (*n* = 10 mothers) exposed mothers. Pearson correlation *r* = 0.55, *P* = 0.012 and corresponding Spearman’s Rank correlation *r* = 0.43, *P* = 0.06. Source data are provided as a Source Data file

To study the relationship between the mothers’ BC exposure during pregnancy and BC accumulation in their placentae, placental tissue of 10 mothers with low and 10 mothers with high residential BC exposure during the whole pregnancy were screened for their BC load. The selection criteria can be found in the Methods section, while the mothers’ residential locations are shown on a CORINE land cover map in Supplementary Fig. 5. BC particles were present in all placentae, with an average (SD) particle count of 0.95 × 10^4^ (0.66 × 10^4^) and 2.09 × 10^4^ (0.96 × 10^4^) particles per mm^3^ for low and high exposed mothers, respectively. Moreover, the placental BC load was positively associated with mothers’ residential BC exposure averaged over the entire pregnancy (Fig. 4, Pearson correlation: *r* = 0.55; *P* = 0.012 and corresponding Spearman’s Rank correlation: r = 0.43; *P* = 0.06). Each 0.5 µg per m^3^ increment in residential BC exposure during pregnancy was associated with +0.45 × 10^4^ particles per mm^3^ (95% CI: 0.11 × 10^4^ to 0.80 × 10^4^; *P* *=* 0.012) or 38.5% higher placental BC load. A note on the size distribution of the identified particles/aggregates can be found as Supplementary Note 1. From the size determination of the identified particles/aggregates it is clear that in the biopsy from each mother, larger particle aggregates, ranging between 1.00 and 9.78 µm, can be found (Supplementary Fig. 1). These particle aggregates consist of various smaller particles which translocated from the mothers’ circulation to distinct locations inside the placental tissue (Supplementary Fig. 2).

### BC load in preterm placentae

**Fig. 5:**
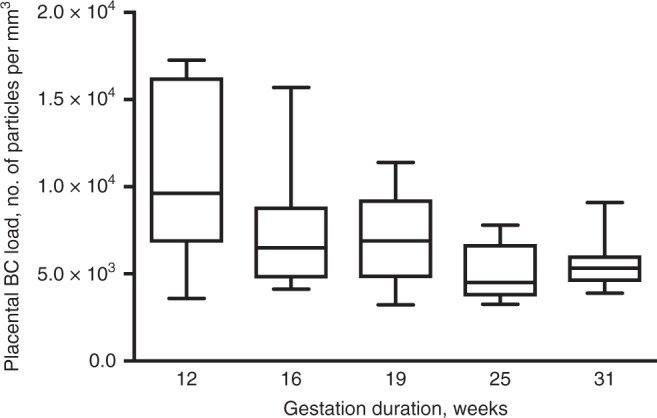
BC load in placentae from spontaneous preterm births (*n* = 5). The whiskers indicate the minimum and maximum value and the box of the box plot illustrates the upper and lower quartile. The median of spreading is marked by a horizontal line within the box. Source data are provided as a Source Data file

To study the ability of BC particles to reach the placenta during early and critical stages of pregnancy, five placentae from spontaneous preterm births were screened for their BC load. The selection criteria can be found in the Methods section. BC particles could be detected in all five placentae (Fig. 5), with a median (SD) particle count between 0.45 × 10^4^ (0.16 × 10^4^) and 0.96 × 10^4^ (0.46 × 10^4^) particles per mm^3^.

## Discussion

In the prospect that ultrafine particles translocate from the mothers’ lungs into the circulation, we screened placentae for their BC loading to investigate whether in a real-life setting particle transfer through the placenta towards the fetal side occurs. Those particles could be identified inside the placenta (Fig. 2) based on the WL generation by the BC particles under femtosecond pulsed illumination (Fig. 1). The signals generated by the identified BC particles under femtosecond pulsed illumination matched precisely the carbonaceous nature of combustion-derived particles (Fig. 3). While the placenta is a heterogeneous organ^26^ and variations in the detected BC particles within one biopsy and between the various biopsies are seen (Supplementary Fig. 4), no significant differences are found between the different biopsies taken at the fetal side of the same placenta. Hence, the screening of one biopsy is sufficient to obtain representative results.

Our results demonstrate that the human placental barrier is not impenetrable for particles. Our observation based on exposure conditions in real-life is in agreement with previously reported ex vivo and in vivo studies studying the placental transfer of various nanoparticles. An ex vivo human perfusion model evidenced that polystyrene particles with a diameter less than 240 nm are able to cross the placenta and hereby even reach the fetal bloodstream^27^. Also, Vidmar et al.^28^ recently demonstrated the translocation of silver nanoparticles to the fetal circulation employing a similar model that mimics the maternal and fetal blood circulation. On the other hand, diesel nanoparticles have been found in maternal red blood cells and plasma, as well as in placental trophoblastic cells from pregnant rabbits exposed to aerosolized diesel exhaust^13^. Our study addressed the gap of human exposure routes of BC particles towards the fetal side of the placenta, although further research is needed, our results suggest that particle transport through placental tissue is indeed possible.

The presence of BC particles could be identified in all screened placentae and a positive association has been found between the placental BC load and the mothers’ residential BC exposure averaged over the entire pregnancy (Fig. 4). We did not have information on the presence of CB-based tattoos of the participating mothers. However, we believe that tattoos are not confounding the association between gestational exposure to particulate air pollution and the placental BC load. First, the largest fraction of the CB particles permanently stay in the dermis between the collagen fibers, while only a minor fraction can be distributed to the lymphatic system and few particles may reach the blood circulation directly^29^. Second, a primary requirement of confounding is that the level of ambient BC particles is correlated with the presence of CB-based tattoos, which is unlikely. Besides in full term placentae, BC particles could also be detected in placentae from pregnancies spontaneously terminated (Fig. 5). The latter shows the presence of BC particles in placental tissue already during the early and vulnerable stages of pregnancy where the fetus is most vulnerable for toxic compounds. However, spontaneous termination of pregnancy may have resulted from complications that could have compromised placental development, and thus its structure and barrier function. Nevertheless, our main analysis is based on full term pregnancies. Further research will have to show whether the particles can cross the placenta and reach the fetus, and if particle translocation is responsible for the observed adverse health effects during early life.

Our current study on the detection of BC particles in placenta includes various strengths, which altogether have led to the reported, important insights. First, our established detection method has several advantages over conventionally used techniques such as light and electron microscopy, which are often employed in this type of epidemiological studies^29,30^. Summarized, our technique does not require extensive sample preparation (e.g., macrophage isolation) nor labeling, the particles can be imaged directly in their biological context (i.e., maternal and fetal side), additional information about the placental structure can be detected simultaneously and, most importantly, it allows specific and sensitive detection of BC particles^19,25^. Second, directly linked to the latter, real-life BC exposures could be measured in the placenta of mothers exposed to relatively low annual ambient BC concentrations (with annual average concentrations ranging between 0.63–2.42 µg per m^3^ in the study area). Despite the low annual ambient BC concentrations in the northern part of Belgium, a Pearson correlation of 0.55 could be found between placental BC load and exposure during pregnancy. Third, we could confirm the carbonaceous nature of the identified BC particles and external contamination of the tissues could be excluded.

In conclusion, our study provides compelling evidence for the presence of BC particles originating from ambient air pollution in human placenta and suggests the direct fetal exposure to those particles during the most susceptible period of life. The evidence of particle translocation to the placenta might be a plausible explanation for the observed detrimental effects of ambient particulate air pollution on fetal development over and beyond the increased maternal systemic inflammation in response to particulate accumulation in the lungs^12,31^.

## Methods

### Study population and sample collection and preparation

The present study on term placentae is executed within the framework of the ENVIR*ON*AGE (ENVIRonmental influence *ON* AGEing in early life) birth cohort^32^. The cohort enrolls mothers giving birth in the East-Limburg Hospital (ZOL; Genk, Belgium) and is approved by the Ethics Committee of Hasselt University and East-Limburg Hospital (EudraCT B37120107805). The study is conducted according to the guidelines laid down in the Declaration of Helsinki. All participating women provided informed written consent. Mothers were asked to fill out a questionnaire to get lifestyle information.

The ambient exposure to BC of the mothers was determined, based on their residential address, using a validated spatial and temporal interpolation method^19,33^. The method uses land cover data obtained from satellite images (CORINE land cover data set) and pollution data of fixed monitoring stations. Coupled with a dispersion model that uses emissions from point sources and line sources, this model chain provides daily exposure values in a high-resolution receptor grid. Overall model performance was evaluated by leave-one-out cross-validation including 16 monitoring points for BC. Validation statistics of the interpolation tool gave a spatiotemporal explained variance of more than 0.74 for BC.

Fresh placentae were collected within 10 min after birth. Biopsies were taken at four standardized sites at the fetal side of the placenta across the middle region, approximately 4 cm away from the umbilical cord and under the chorio-amniotic membrane. The order of the biopsies was clockwise starting at the main blood vessel. Also, one biopsy is taken at the maternal side of the placenta at the equivalent position of biopsy 1 of the fetal side. Accordingly, the biopsies taken at the sides facing towards the fetus and mother are defined as the fetal and maternal side of the placenta, respectively.

The intervariability and intravariability between and within biopsies were assessed using placental tissue from three randomly selected, non-smoking mothers, with average residential BC exposure (between 0.96 and 1.32 µg per m^3^).

To evaluate the correlation between the BC exposure of mothers (all non-smokers) during pregnancy and accumulation of BC in placentae, 10 mothers with high residential BC exposure and 10 mothers with low residential BC exposure during pregnancy were selected from the ENVIR*ON*AGE biobank. High residential BC exposure during pregnancy was defined as: (i) entire pregnancy and third trimester of pregnancy exposure to residential BC ≥ 75th percentile (1.70 µg per m³ and 2.42 µg per m³, respectively), and (ii) residential proximity to a major road ≤ 500 m. Low residential BC exposure during pregnancy was defined as: (i) entire pregnancy and third trimester of pregnancy exposure to residential BC ≤ 25th percentile (0.96 µg per m³ and 0.63 µg per m³, respectively), and (ii) residential proximity to major road >500 m.

Biopsies of placental tissue from spontaneous preterm births were collected at the East-Limburg Hospital (ZOL; Genk, Belgium). Five biobanked placentae of mothers with spontaneous termination of pregnancy between 12 and 31 weeks of gestation were randomly selected but taking into account the following criteria: (i) non-smoker, (ii) avoiding possible complications that can cause autolysis (mors in utero) or disturb the histological image (infections), and (iii) best possible spread in gestation time, i.e., pregnancy termination at 12, 16, 19, 25, and 31 weeks. The cases were handled strictly anonymously. Accordingly, no personal information is available except for the inclusion and exclusion criteria and, thus, the residential exposure to ambient air pollution is unknown. The use of these tissues for the detection of BC particles was approved by the Ethics Committee of Hasselt University and East-Limburg Hospital (EudraCT B371201938875). Since the employed samples were biobanked, this specific study is not covered by the law of 7 May 2004 on experiments on the human person. Hence, no written consent was needed according to the Ethical Committees. To study the BC loading in the preterm placentae the available biopsy was screened by imaging three regions within five different sections taken in the middle of the tissue (*n* = 15 images).

Placental biopsies were fixed in formaldehyde for minimal 24 h and paraffin embedded. 4 µm sections were cut using a microtome (Leica Microsystems, UK) and mounted between histological glass slides. To preclude any particulate contamination, particle-free instruments and sample holders were used and all samples were handled in a clean room with filtered air (Genano 310/OY, Finland).

### Experimental protocol for BC detection in placentae

BC particles naturally present in the placenta were detected using a specific and sensitive detection technique based on the non-incandescence-related WL generation of the particles under femtosecond illumination as published before^19,25^. Images of the placental sections were collected at room temperature using a Zeiss LSM 510 (Carl Zeiss, Jena, Germany) equipped with a two-photon femtosecond pulsed laser (810 nm, 150 fs, 80 MHz, MaiTai DeepSee, Spectra-Physics, USA) tuned to a central wavelength of 810 nm with 5 or 10 mW radiant power on average at the sample position using a 10×/0.3 objective (Plan-Neofluar 10×/0.3, Carl Zeiss). WL emission of the BC particles was acquired in the non-descanned mode after spectral separation and emission filtering using 400–410 nm and 450–650 nm BP filters. By employing these two emission filters, the SHG from the placental collagen type I and TPAF of the placental components are collected in the corresponding images. The resulting tile scans had a field of view of 9000 × 9000 µm^2^ containing 100 images with a pixel size of 0.694 µm and were recorded with a 2.51 µs pixel dwell time. The spatial resolution of the system in the configuration that the measurements were performed (i.e., 10 × /0.3 objective, 810 nm excitation, identical settings): *w*_x_ = *w*_y_ = 1.44 µm and *w*_z_ = 14.8 µm defined as the sizes of the point spread function in the XY-plane (radius of Airy-disk) and along the optical axis (1/e-thickness), respectively. The images were acquired by ZEN Black 2.0 software (Zeiss).

To count the number of BC particles in the tile scans of each placental section, an automated and customized Matlab program (Matlab 2010, Mathworks, The Netherlands) was used. First, a peak-find algorithm detects pixels above a certain threshold value. Here, threshold values of 0.5% and 45% lower than the highest pixel intensity value of the TPAF and SHG image, respectively, were chosen. These thresholds resulted in highly reproducible values, which were checked manually using Fiji (ImageJ v2.0, Open source software, http://fiji.sc/Fiji). Next, the detected pixels of both images are compared and only the matching ones are used to generate the output image and metrics. In addition, the effectively imaged placental area was determined from the TPAF image using Fiji and the focal volume based on the point spread function of the optical system. Finally, the total relative number, i.e., the number of detected BC particles per cubic millimeter imaged placenta, was defined.

The customized Matlab program is made available upon reasonable request directed to the corresponding author.

### Validation experiments of WL from BC in placentae

Validation experiments were performed using a Zeiss LSM 880 (Carl Zeiss, Jena, Germany) and 40×/1.1 water immersion objective (LD C-Apochromat 40×/1.1 W Korr UV-Vis-IR, Carl Zeiss). This setup was used as it allows accurate detection of the emission fingerprint and time correlated single photon counting of the BC particles in placental tissue. All settings were kept identical compared to the measurements performed on the LSM 510 setup unless stated otherwise.

Approximately, 60 images with a pixel size of 0.297 × 0.297 × 0.500 µm^3^ were acquired throughout the placental section using a pixel dwell time of 4.1 µs. In total, a volume of 300 × 300 × 30 µm^3^ was imaged. Orthogonal XZ-projection and YZ-projection were made using Fiji.

The emission fingerprints of the BC particles inside the placental tissue sections and TPAF from the placental cells were collected under femtosecond pulsed illumination. Note, for this specific experiment, the gain and laser power were changed to avoid saturation of the emission signal in order to be able to observe the trend of the WL signal over all wavelengths. After spectral separation, the emitted signals ranging between 410–650 nm were collected at an interval of 9 nm using the QUASAR thirty-two channel GaASP spectral detector of the LSM 880 system. The resulting 1024 × 1024 lambda image with a pixel size of 0.104 µm was detected with a pixel dwell time of 2.05 µs. As a reference, the emission fingerprint of commercially available carbon black nanoparticles (US Research Nanomaterials, USA) was recorded using identical settings.

Following femtosecond illumination, the temporal responses of the emitted signals originating from the BC particles in the placental tissue and from the placental cells were detected using the BiG.2 GaASP detector of the LSM 880 system. The detector was connected to an SPC 830 card (Becker and Hickl, Germany) that was synchronized to the pulse train of the MaiTai DeepSee laser. Recordings of 256 × 256 images with a pixel size of 0.346 µm were acquired using a pixel dwell time of 8.19 µs. The instrument response function was determined by detecting the response (IRF) of the laser pulse using potassium dihydrogen phosphate crystals under identical conditions. The IRF value was used in the analysis of all measurements for curve fitting. As a reference, the temporal response of commercially available carbon black particles was recorded employing the same settings. All time-correlated single photon counting measurements were captured and analyzed using the SPCM 9.80 and SPCImage 7.3 software (Becker and Hickl), respectively.

### Screening of placental tissue for BC load

Both the intravariability and intervariability in BC loading of the placental biopsies were evaluated. The intravariability (within one biopsy) was examined by screening three regions of 10 × 10 images within five different sections taken in the middle of the examined biopsy (*n* = 15 images per biopsy). On the other hand, the intervariability (between the four fetal biopsies) was studied by measuring the BC load in three regions of 10 × 10 images within five different sections taken in the middle of the examined biopsy (*n* = 60 images per mother). The intervariability was solely assessed between the four fetal biopsies since there is already an existing variability between the fetal and maternal biopsies.

To evaluate the BC loading in the placentae of 10 low and 10 high exposed mothers, one biopsy was examined. More specifically, biopsy number 2 was selected with the exception of the following cases: (i) no or too little tissue was available, or (ii) background signal of blood was too high. In the latter cases, biopsy number 3 was chosen. The BC load was measured in three regions within five different sections taken in the middle of the biopsy (*n* = 15 images).

To study the BC loading in the preterm placentae the available biopsy was screened by imaging three regions within five different sections taken in the middle of the tissue (*n* = 15 images).

### Ultrastructural analysis

Following fixation in 2% glutaraldehyde, the biopsies were gently rinsed and postfixed in 2% osmium tetroxide for 1 h. Subsequently, the biopsies were put through a dehydrating series of graded concentrations of acetone and impregnated overnight in a rotator with acetone:spurr (1:1) (Spurr Embedding Kit, Electron Microscopy Sciences). Next, the samples are placed into molds and fresh spurr solution is added followed by polymerization for 24–36 h at 70 °C. Ultra-thin sections (60 nm) were mounted on 0.7% formvar-coated copper grids and examined in a Philips EM 208 transmission electron microscope operated at 60 kV. Digital images were captured using a Morada camera system and analyzed using SIS analysis software (Germany).

### Statistical analysis

All data are represented as means ± standard deviation and were analyzed using the commercially available software Graphpad (Graphpad Prism 6, Graphpad Software Inc., USA) and JMP (JMP Pro 12, SAS Institute Inc., USA). On the intravariability and intervariability data, a two-tailed analysis of variance (ANOVA) was performed followed by the Tukey posttest. To assess the relation between the BC exposure of mothers during pregnancy and accumulation of BC in placentae, the Pearson correlation coefficients were determined. We used the nonparametic Spearman’s Rank test to confirm the results.

### Reporting summary

Further information on research design is available in the Nature Research Reporting Summary linked to this article.

## Supplementary information

Supplementary Information

Reporting Summary

Source Data

## Data Availability

The data that support the findings of this study are not publicly available as they contain information that could compromise research participant privacy, but are available from the corresponding author (T.S.N.) upon reasonable request. The source data underlying Figs. 3b, c, 4, and 5 and Supplementary Figs 1 and 4a-c are provided as a Source Data file.
