# Supplementary material for: Ambient black carbon particles reach the fetal side of human placenta
Source: Nat Commun. 2019 Sep 17;10:3866. doi: 10.1038/s41467-019-11654-3 (PMC6748955; doi:10.1038/s41467-019-11654-3)
Supplement: Supplementary file 1 — Supplementary Information [file 41467_2019_11654_MOESM1_ESM.docx]

**Supplementary Information**

**Ambient Black Carbon Particles Reach the Fetal Side of Human Placenta**

Hannelore Bové^1,2,3,§^, Eva Bongaerts^1,§^, Eli Slenders^2^, Esmée M. Bijnens^1^, Nelly D. Saenen^1^, Wilfried Gyselaers^4^, Peter Van Eyken^4^, Michelle Plusquin^1^, Maarten B. J. Roeffaers^3^, Marcel Ameloot^2^, Tim S. Nawrot^*,1,5^

*^1^Centre for Environmental Sciences, Hasselt University, Agoralaan Building D, 3590 Diepenbeek, Belgium*

*^2^Biomedical Research Institute, Hasselt University, Agoralaan Building C, 3590 Diepenbeek, Belgium*

*^3^Centre for Surface Chemistry and Catalysis, KU Leuven, Celestijnenlaan 200F - box 2461, 3001 Leuven, Belgium*

*^4^ Department of Obstetrics, East-Limburg Hospital, Schiepse Bos 6, 3600 Genk, Belgium*

*^5^Department of Public Health and Primary Care, KU Leuven, Herestraat 49 - box 706, 3000 Leuven, Belgium.*

*^§^ These authors contributed equally.*

**Supplementary Figures**

**Supplementary Figure 1.** Size distribution (mean ± standard deviation; n=15 analyzed images) of the BC particle aggregates found in the screened term placentae (n=20 placentae) using white-light detection under femtosecond pulsed illumination. Source data are provided as a Source Data file.

***
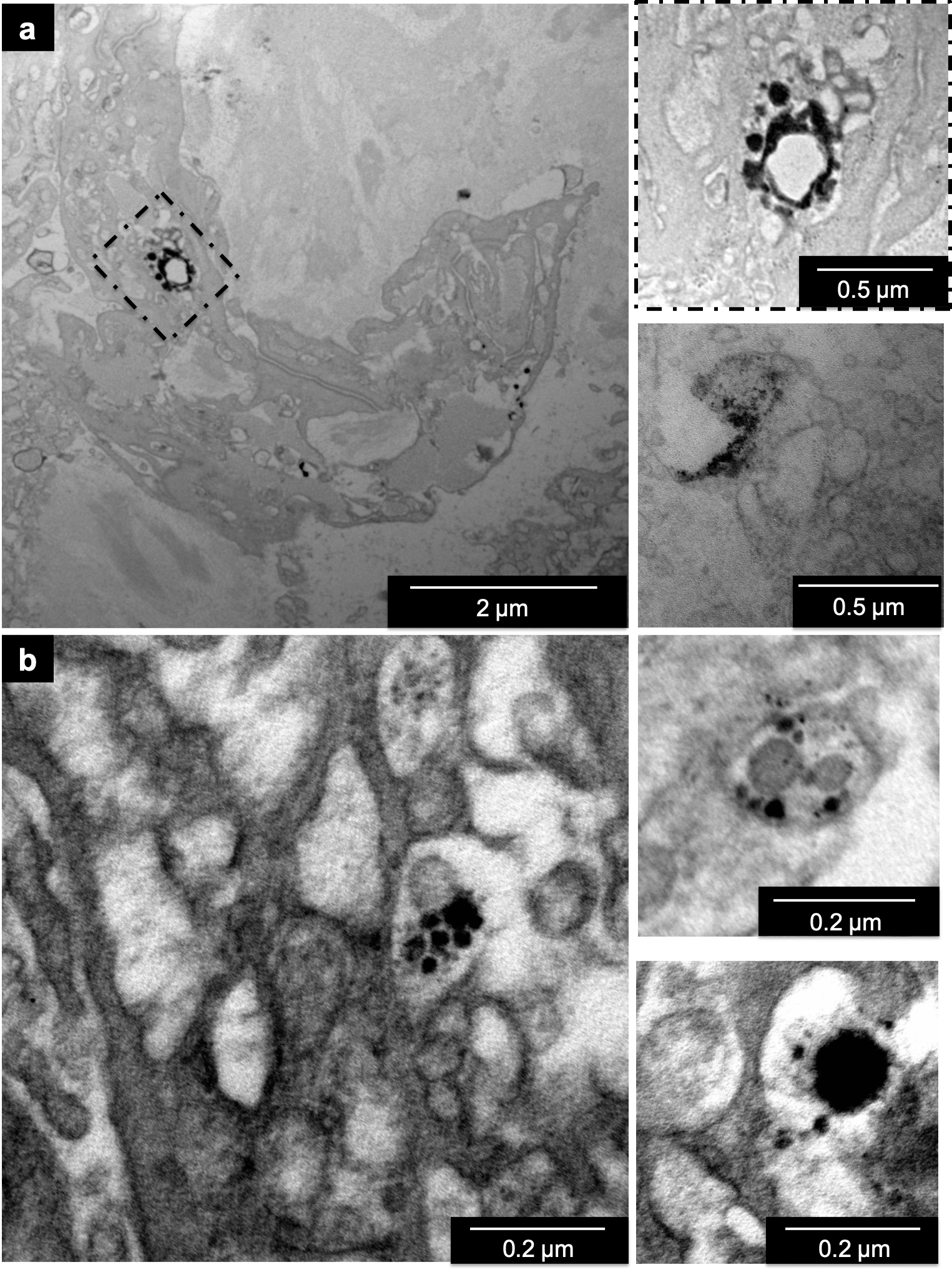
***

**Supplementary Figure 2.** Transmission electron microscopy imaging of BC particles inside placenta thin sections of two **a-b** high exposed mothers.

**
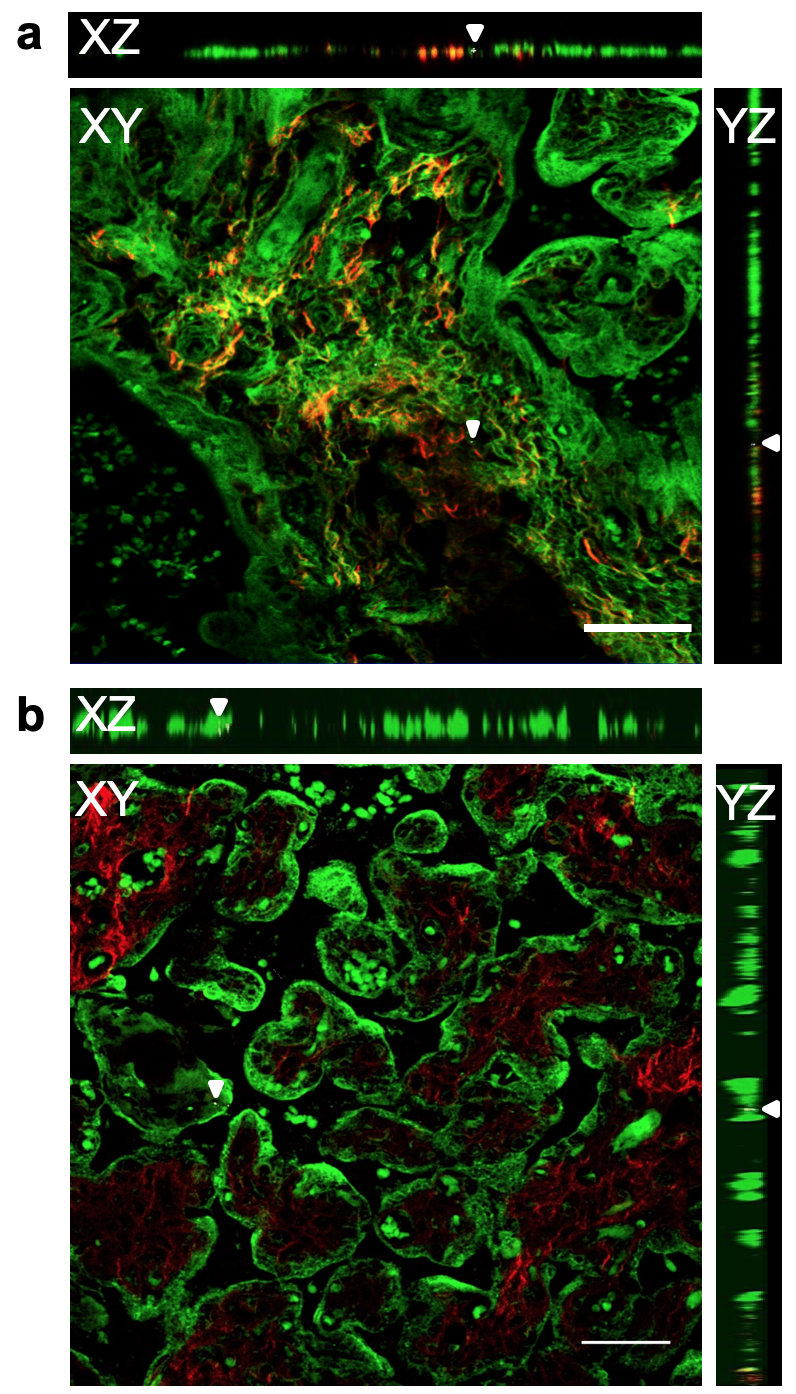
**

**Supplementary Figure 3.** XY-images acquired throughout placental sections in the z-direction and corresponding orthogonal XZ- and YZ-projections showing BC particles (white and indicated by white arrowheads) inside the tissue (red and green). **a** Data as presented in the main text but showing another embedded BC particle. **b** Data from another mother. Scale bars: 50 *µ*m.

**
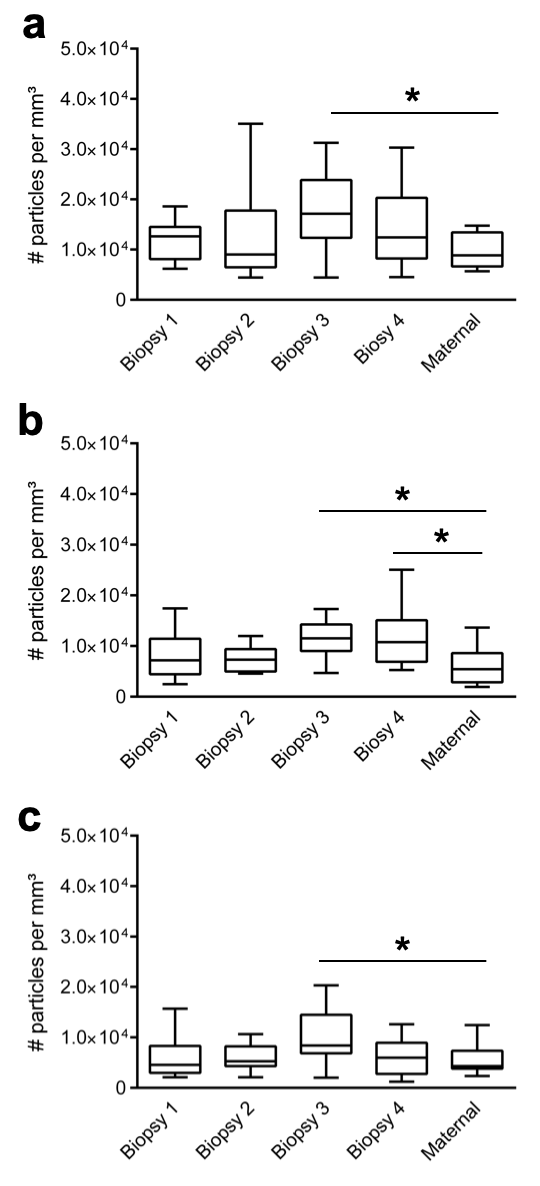
**

**Supplementary Figure 4.** Intravariability (n=15 images per biopsy) and intervariability (n=60 images per mother) of the placental tissue including four biopsies taken at the fetal side and one at the maternal side of the placenta from three different mothers **a-c**. The whiskers indicate the minimum and maximum value and the box of the box plot illustrates the upper and lower quartile. The median of spreading is marker by a horizontal line within the box. Significant differences between the different biopsies were assessed by one-way ANOVA test (multiple comparisons) followed by Tukey test. *P*-value: **P*≤0.05. Source data are provided as a Source Data file.

**
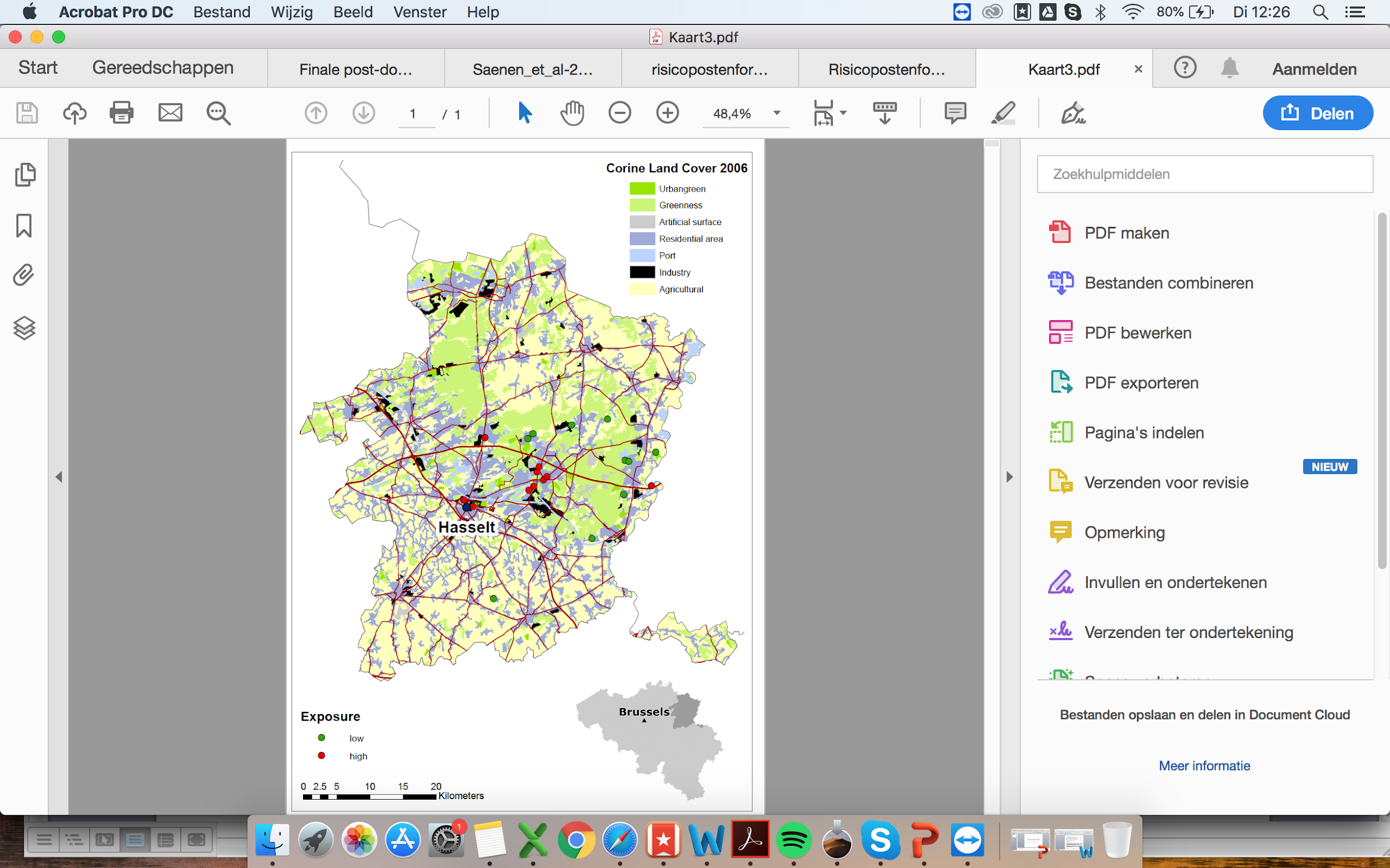
**

**Supplementary Figure 5.** The residential addresses of the mothers are located in the north-east of Belgium. Red dots represent the residences of the mothers with a high residential BC exposure during their whole pregnancy, whereas the green dots represent residences with a low exposure. Major roads are indicated as red lines on the map.

**Supplementary Notes**

**Supplementary Note 1: size distribution of identified BC particles/aggregates in placental tissue**

Regarding to the size determination of the BC particles/aggregates, it should be noted that the employed method is optical-based. The measured optical point spread function (PSF) of the system (810 nm, 0.3 NA) is 1.44 *µ*m (radius of Airy-disk). The PSF puts a lower limit to the particle size that can be estimated. Hence, the size of particles with diameters smaller than 2.88 *µ*m (diameter of Airy-disk) cannot be determined accurately.

Nonetheless, we analyzed the sizes of the particle aggregates of the screened placental biopsies from the 10 low and 10 high exposed mothers. The results are summarized in Supplementary Figure 1. From the analysis it is clear that in the biopsy from each mother, larger particle aggregates, ranging between 1.00 and 9.78 *µm,* can be found. Most likely, these particle aggregates consist of various smaller particles, which translocated from the mothers’ circulation to distinct locations inside the placental tissue.

Since the optical resolution depends on the numerical aperture of the employed objective, one can argue that an objective with higher NA can be employed to gain better insights into the size distribution of the particles. However, screening tissues with a high NA objective would take too much time. Moreover, even with a high NA objective the optical resolution still stands and particles with sizes below 250 nm cannot be determined.

Therefore, transmission electron microscopy was used to investigate the size distribution of the BC particles in placental thin sections of two high exposed mothers. The representative images in Supplementary Figure 2 show that also smaller particles are present inside the placental tissue. Moreover, it is clear that the particles tend to aggregate inside cellular structures.
