# Supplementary material for: Ambient black carbon particles reach the fetal side of human placenta
Source: Nat Commun. 2019 Sep 17;10:3866. doi: 10.1038/s41467-019-11654-3 (PMC6748955; doi:10.1038/s41467-019-11654-3)
Supplement: Supplementary file 2 — Reporting Summary [file 41467_2019_11654_MOESM2_ESM.pdf]

nature research

Corresponding author(s): Tim S. Nawrot

Last updated by author(s): Jun 24, 2019

Reporting Summary

Nature Research wishes to improve the reproducibility of the work that we publish. This form provides structure for consistency and transparency in reporting. For further information on Nature Research policies, see Authors & Referees and the Editorial Policy Checklist.

Statistics

For all statistical analyses, confirm that the following items are present in the figure legend, table legend, main text, or Methods section.

n/a	Confirmed	
☐	☒	The exact sample size (*n*) for each experimental group/condition, given as a discrete number and unit of measurement
☐	☒	A statement on whether measurements were taken from distinct samples or whether the same sample was measured repeatedly
☐	☒	The statistical test(s) used AND whether they are one- or two-sided<br>*Only common tests should be described solely by name; describe more complex techniques in the Methods section.*
☒	☐	A description of all covariates tested
☒	☐	A description of any assumptions or corrections, such as tests of normality and adjustment for multiple comparisons
☐	☒	A full description of the statistical parameters including central tendency (e.g. means) or other basic estimates (e.g. regression coefficient) AND variation (e.g. standard deviation) or associated estimates of uncertainty (e.g. confidence intervals)
☐	☒	For null hypothesis testing, the test statistic (e.g. *F*, *t*, *r*) with confidence intervals, effect sizes, degrees of freedom and *P* value noted<br>*Give P values as exact values whenever suitable.*
☒	☐	For Bayesian analysis, information on the choice of priors and Markov chain Monte Carlo settings
☒	☐	For hierarchical and complex designs, identification of the appropriate level for tests and full reporting of outcomes
☒	☐	Estimates of effect sizes (e.g. Cohen's *d*, Pearson's *r*), indicating how they were calculated

Our web collection on statistics for biologists contains articles on many of the points above.

Software and code

Policy information about availability of computer code

Data collection	The tile scans of placenta sections were acquired by ZEN Black 2.0 software (Zeiss). All time-correlated single photon counting measurements were collected using the SPCM 9.80 and SPCImage 7.3 software (Becker and Hickl).
Data analysis	An automated and customized Matlab program (Matlab 2010, Mathworks) was used to count the number of BC particles in the tile scans of each placenta section. The effectively imaged placenta area was determined using Fiji (ImageJ v2.0, Open source software). All data was analyzed using Graphpad software (Graphpad Prism 6, Graphpad Software Inc.) and JMP (JMP Pro 12, SAS Institute Inc.)<br>SIS analysis software was used to analyze the transmission electron microscopy images.

For manuscripts utilizing custom algorithms or software that are central to the research but not yet described in published literature, software must be made available to editors/reviewers. We strongly encourage code deposition in a community repository (e.g. GitHub). See the Nature Research guidelines for submitting code & software for further information.

Data

Policy information about availability of data

All manuscripts must include a data availability statement. This statement should provide the following information, where applicable:

- Accession codes, unique identifiers, or web links for publicly available datasets
- A list of figures that have associated raw data
- A description of any restrictions on data availability

The data that support the findings of this study are not publicly available as they contain information that could compromise research participant privacy but are available from the corresponding author (T.S.N) on reasonable request. The source data underlying Figures 3b-C, 4 and 5 and Supplmentary Figures 1 and 4a-C are provided as a Source Data file.

Field-specific reporting

Please select the one below that is the best fit for your research. If you are not sure, read the appropriate sections before making your selection.

☐ Life sciences ☐ Behavioural & social sciences ☒ Ecological, evolutionary & environmental sciences

For a reference copy of the document with all sections, see nature.com/documents/nr-reporting-summary-flat.pdf

Ecological, evolutionary & environmental sciences study design

All studies must disclose on these points even when the disclosure is negative.

Study description	Combustion-derived particulate matter, namely black carbon, is identified in placenta tissue using a non-incandescence related white-light generation under femtosecond pulsed illumination.
Research sample	The term placentae are obtained within the framework of the ENVIRONAGE (ENVIRonmental influence ON AGEing in early life) birth cohort which enrolls mothers giving birth in the South-East-Limburg Hospital (Genk, Belgium). The preterm placentae are obtained from the biobank from the South-East-Limburg Hospital (Genk, Belgium) without the need for informed consent. The studies are approved by the Ethics committee of Hasselt University and South-East-Limburg Hospital and conducted according to the guidelines laid down in the Declaration of Helsinki.
Sampling strategy	To evaluate the correlation between the BC exposure of mothers during pregnancy and accumulation of BC in placentas, 10 mothers with high residential BC exposure and 10 mothers with low residential BC exposure were selected from the ENVIRONAGE biobank. In addition, the inter- and intravariability between and within biopsies was assessed using placenta tissue from three randomly selected mothers with moderate residential exposure. Furthermore, placental tissue from spontaneous preterm births of five individuals were collected. No sample-size calculation was performed and in total 28 placenta's were screened for their black carbon load. A total of 28 placenta's was chosen in this study to confirm the presence of black carbon particles in the placenta, both at term and during earlier stages of pregnancy.
Data collection	Black carbon particles, naturally present in the placenta were detected using a specific and sensitive detection technique based on the non-incandescence-related white-light generation of the particles under femtosecond pulsed illumination. The data is collected by Ev.B. and H.B using a confocal microscope and commercial software.
Timing and spatial scale	In this analysis, placentas from 10 mothers with high residential BC exposure and 10 mothers with low residential BC exposure were selected. The high-exposed individuals were recruited between November 20 2011 and April 12 2015, whereas the low-exposed individuals were recruited between October 3 2014 and October 6 2016. The three randomly selected participating mothers, to assess inter- and intravariability, were recruited between January 24 and March 1 2014. The preterm placental biopsies were collected in 2017 and 2018. Detection of black carbon particles naturally present in these placentas was performed between Augustus 17 and February 15 2019.
Data exclusions	Data from placental tissue collected from smoking mothers' were excluded to prevent detection of BC particles originating from maternal smoking and not ambient air pollution.
Reproducibility	All attempts to repeat the measurements were successful.
Randomization	High residential BC exposure during pregnancy for the 10 high exposed individuals was defined as: (i) entire pregnancy and third trimester of pregnancy exposure to residential BC larger or equal to the 75th percentile (1.41 µg/m^3 and 1.50 µg/m^3, respectively), and (ii) residential proximity to a major road smaller or equal to 500 m. Low residential BC exposure during pregnancy was defined as: (i) entire pregnancy and third trimester of pregnancy exposure to residential BC smaller or equal to the 25th percentile (0.97 µg/m^3 and 0.90 µg/m^3, respectively), and (ii) residential proximity to a major road is larger than 500 m. The five individuals with spontaneous preterm births were randomly selected but taking into account the following criteria: (i) non-smoker, (ii) avoiding possible complications that can cause autolysis or disturb the histological image, and (iii) best possible spread in gestation time. The inter- and intravariability between and within placental biopsies was assessed using placenta tissue from three randomly selected non-smoking mothers. The three individuals used to determine intra- en intervariability were randomly allocated.
Blinding	A specific study number is assigned to the participating mother-child pairs to assure their privacy and to exclude biasing.

Did the study involve field work? ☐ Yes ☒ No

Reporting for specific materials, systems and methods

We require information from authors about some types of materials, experimental systems and methods used in many studies. Here, indicate whether each material, system or method listed is relevant to your study. If you are not sure if a list item applies to your research, read the appropriate section before selecting a response.

Materials & experimental systems

n/a	Involved in the study	
☒	☐	Antibodies
☒	☐	Eukaryotic cell lines
☒	☐	Palaeontology
☒	☐	Animals and other organisms
☐	☒	Human research participants
☒	☐	Clinical data

Methods

n/a	Involved in the study	
☒	☐	ChIP-seq
☒	☐	Flow cytometry
☒	☐	MRI-based neuroimaging

Human research participants

Policy information about studies involving human research participants

Population characteristics

Demographic and lifestyle characteristics of the ENVIRONAGE birth cohort participants are similar to those of the Flemish birth register of all births between 2002 and 2011 in the northern part of Belgium. Hence, our mother-child cohort is representative for the gestational segment of the population in Flanders. A paper on the cohort characteristics is available. To evaluate the correlation between the BC exposure of mothers (all non- smokers) during pregnancy and accumulation of BC in placentae, 10 mothers with high residential BC exposure and 10 mothers with low residential BC exposure during pregnancy were selected from the ENVIRONAGE biobank. High residential BC exposure during pregnancy was defined as: (i) entire pregnancy and third trimester of pregnancy exposure to residential BC ≥ 75th percentile (1.70 μg per m^3 and 2.42 μg per m^3, respectively), and (ii) residential proximity to a major road ≤ 500 m. Low residential BC exposure during pregnancy was defined as: (i) entire pregnancy and third trimester of pregnancy exposure to residential BC ≤ 25th percentile (0.96 μg per m^3 and 0.63 μg per m^3, respectively), and (ii) residential proximity to major road > 500 m.

Five biobanked placentae of mothers with spontaneous termination of pregnancy between 12 and 31 weeks of gestation were randomly selected but taking into account the following criteria: (i) non-smoker, (ii) avoiding possible complications that can cause autolysis (mors in utero) or disturb the histological image (infections), and (iii) best possible spread in gestation time, i.e. pregnancy termination at 12, 16, 19, 25 and 31 weeks. The cases were handled strictly anonymously. Accordingly, no personal information is available except for the inclusion and exclusion criteria and, thus, the residential exposure to ambient air pollution is unknown.

Recruitment

Mother-newborn pairs are recruited when they arrive for delivery at the East-Limburg Hospital in Genk (Belgium) following procedures approved by the Ethical Committee of Hasselt University and the East-Limburg Hospital. Mothers without planned caesarean section and able to fill out a Dutch language questionnaire are eligible for the cohort.

The preterm placental samples are derived from the biobank of the East-Limburg Hospital (Genk, Belgium).

Ethics oversight

The cohort study is approved by the Ethics committee of Hasselt University and South-East-Limburg Hospital (EudraCT B37120107805, Belgium). All participating women provided informed written consent.

The use of the preterm placental tissues for the detection of BC particles was approved by the Ethics Committee of Hasselt University and East-Limburg Hospital (EudraCT B371201938875). Since the employed samples were biobanked, this specific study is not covered by the law of 7 May 2004 on experiments on the human person. Hence, no written consent was needed according to the Ethical Committees.

Note that full information on the approval of the study protocol must also be provided in the manuscript.
